# Investigation of Lupeol as Anti-Melanoma Agent: An In Vitro-In Ovo Perspective

**DOI:** 10.3390/curroncol28060425

**Published:** 2021-12-02

**Authors:** Flavia Bociort, Ioana Gabriela Macasoi, Iasmina Marcovici, Andrei Motoc, Cristina Grosu, Iulia Pinzaru, Crina Petean, Stefana Avram, Cristina Adriana Dehelean

**Affiliations:** 1 Faculty of Medicine, “Victor Babes” University of Medicine and Pharmacy Timisoara, Eftimie Murgu Square No. 2, 300041 Timisoara, Romania; flavia.bociort@umft.ro (F.B.); amotoc@umft.ro (A.M.); 2 Faculty of Pharmacy, “Victor Babes” University of Medicine and Pharmacy Timisoara, Eftimie Murgu Square No. 2, 300041 Timisoara, Romania; macasoi.ioana@umft.ro (I.G.M.); cristina.grosu@umft.ro (C.G.); iuliapinzaru@umft.ro (I.P.); crina.petean@umft.ro (C.P.); stefana.avram@umft.ro (S.A.); cadehelean@umft.ro (C.A.D.); 3 Research Center for Pharmaco-Toxicological Evaluations, “Victor Babes” University of Medicine and Pharmacy Timisoara, Eftimie Murgu Square No. 2, 300041 Timisoara, Romania

**Keywords:** lupeol, skin cancer, malignant melanoma cells, angiogenesis

## Abstract

Malignant melanoma (MM) represents the most life-threatening skin cancer worldwide, with a narrow and inefficient chemotherapeutic arsenal available in advanced disease stages. Lupeol (LUP) is a triterpenoid-type phytochemical possessing a broad spectrum of pharmacological properties, including a potent anticancer effect against several neoplasms (e.g., colorectal, lung, and liver). However, its potential as an anti-melanoma agent has been investigated to a lesser extent. The current study focused on exploring the impact of LUP against two human MM cell lines (A375 and RPMI-7951) in terms of cell viability, confluence, morphology, cytoskeletal distribution, nuclear aspect, and migration. Additionally, the in ovo antiangiogenic effect has been also examined. The in vitro results indicated concentration-dependent and selective cytotoxicity against both MM cell lines, with estimated IC_50_ values of 66.59 ± 2.20 for A375, and 45.54 ± 1.48 for RPMI-7951, respectively, accompanied by a reduced cell confluence, apoptosis-specific nuclear features, reorganization of cytoskeletal components, and inhibited cell migration. In ovo, LUP interfered with the process of angiogenesis by reducing the formation of neovascularization. Despite the potential anti-melanoma effect illustrated in our in vitro-in ovo study, further investigations are required to elucidate the underlying LUP-induced effects in A375 and RPMI-7951 MM cells.

## 1. Introduction

Malignant melanoma (MM) is described as the tumor arising from the neoplastic transformation of melanin-producing cells—the melanocytes [1], and the leading cause of skin cancer-related deaths worldwide [2] despite its rareness [3]. The pathogenesis of MM is multifactorial [4]. However, cutaneous MM (CMM) development is primarily the consequence of skin exposure to environmental carcinogens, such as ultraviolet radiation (UVR) from natural or artificial sources [3]. Other noxious factors leading to CMM occurrence include gene mutations (e.g., CDKN2A, p53, RB1, BRAF), immunosuppression, oxidative stress due to reactive oxygen species (ROS) generation, and inflammatory responses [4,5]. Treatment strategies vary from surgical resection to chemo-, radio-, and immuno-therapies, being highly dependent on the tumor stage, location, and genetics. Chemotherapy (e.g., dacarbazine) was the first-line option for advanced melanoma management [6,7,8], but due to severe adverse events, drug resistance, and lack of survival benefit, the treatment success rate has been considerably lowered [9,10,11]. The management of metastatic MM has significantly progressed with the emergence of targeted therapy and immune checkpoint inhibitors. However, each therapy option presents several limitations. Targeted therapy (e.g., vemurafenib, dabrafenib) has been associated with a high response rate, but also with short-term therapeutic benefits and rapid development of tumor resistance (within several months). Conversely, the treatment with immune checkpoint inhibitors (e.g., ipilimumab, pembrolizumab, nivolumab, atezolizumab) generates a superior overall survival among patients, but a lower response rate [11,12]. To overcome the current difficulties enquired in the treatment of advanced MM, improved therapeutic strategies are deeply required.

Medicinal plants serve as excellent sources for drug discovery and development due to their richness in secondary metabolites displaying a wide structural and pharmacological diversity that favors this process [13]. Natural compounds are extensively investigated as complementary or alternative choices in cancer prevention or chemotherapy [14]. In regard to MM, plant constituents (e.g., terpenoids, flavonoids) [7,15] generally serve as potent anti-melanoma agents by inhibiting the proliferation, migration, and metastasis of melanoma cells [9]. Lupeol (LUP) is a dietary pentacyclic lupane-type triterpenoid found in various foods, such as fruits and vegetables, as well as medicinal plants. From a therapeutic point of view, the latest research of the pharmacological properties of LUP suggests a broad portfolio of biological effects, such as antioxidant, anti-inflammatory, antimicrobial, and skin protective [16,17]. In addition, the anticancer activity of LUP has been demonstrated against several malignancies, such as colorectal, lung, and liver neoplasms [18,19,20]. However, the data on the potential of LUP in malignant melanoma treatment remain scarce.

The current study aims at providing an in vitro-in ovo insight into the anti-proliferative, anti-metastatic, and antiangiogenic properties of LUP as a potential chemotherapeutic agent in malignant melanoma management.

## 2. Materials and Methods

### 2.1. Reagents

Lupeol, trypsin-EDTA solution, phosphate saline buffer (PBS), dimethyl sulfoxide (DMSO), fetal calf serum (FCS), penicillin/streptomycin, and MTT [3-(4,5-dimethylthiazol-2-yl)-2,5-diphenyltetrazolium bromide] reagent were purchased from Sigma Aldrich, Merck KgaA (Darmstadt, Germany). The cell culture media, Dulbecco’s Modified Eagle Medium (DMEM; ATCC^®^ 30-2002™) and Eagle’s Minimum Essential Medium (EMEM; ATCC^®^ 30-2003™), were purchased from ATCC (American Type Cell Collection, Lomianki, Poland). Hoechst 33342 reagent was obtained from Invitrogen by Thermo Fisher Scientific (Waltham, MA, USA). All reagents were analytically pure and proper for cell culture use.

### 2.2. Cell Culture

The anti-melanoma effect of LUP was evaluated on two human MM cell lines provided by ATCC as frozen vials—A375 (CRL-1619™), and RPMI-7951 (HTB-66™). The biosafety of LUP was assessed on two healthy skin cell types (human keratinocytes—HaCaT, and human fibroblasts—1BR3) provided as frozen vials by CLS Cell Lines Service GmbH (Eppelheim, Germany), and the European Collection of Authenticated Cell Cultures (ECACC, Salisbury, UK), respectively. Each cell line was cultured in its specific growth medium (A375 and HaCaT in DMEM, and RPMI-7951 in EMEM) containing 10% FCS and 1% penicillin (100 U/mL)-streptomycin (100 µg/mL) mixture. The 1BR3 cells were cultured in EMEM medium supplemented with 15% FCS and antibiotics. The cells were incubated in standard conditions (37 °C, and 5% CO_2_) during the experiments.

### 2.3. Cellular Viability and Morphology Assessment

The cell viability was assessed by applying the MTT technique. Briefly, A375, RPMI-7951, HaCaT, and 1BR3 cells were cultured in 96-well plates (10^4^ cells/well) and stimulated with five concentrations (10, 20, 30, 40, and 50 µM) of LUP. DMSO was used for the preparation of the LUP stock solution. After 24 h of treatment, fresh media (100 µL) and MTT reagent (10 µL) were added in each well, and the plates were incubated for 3 h at 37 °C. The final steps included: addition of the solubilization solution (100 µL/well), incubation of the plates at room temperature for 30 min, protected from light, and absorbance measurement at two wavelengths (570 and 630 nm) using Cytation 5 (BioTek Instruments Inc., Winooski, VT, USA). In order to verify the potential impact of LUP on A375 and RPMI-7951 cells’ morphology and confluence, a microscopic evaluation was performed by photographing the cells under bright field illumination at the end of the 24 h stimulation period using Cytation 1 (BioTek Instruments Inc., Winooski, VT, USA). The pictures were analyzed using the Gen5™ Microplate Data Collection and Analysis Software (BioTek Instruments Inc., Winooski, VT, USA).

### 2.4. Nuclear Morphology Evaluation

To evaluate the potential toxicity of LUP at the nuclear level, Hoechst 33342 staining was applied according to the manufacturer’s recommendations. In brief, the A375 and RPMI-7951 cells were seeded in 12-well plates (10^5^ cells/well) and treated with three increasing concentrations (10, 30, and 50 µM) of LUP. After 24 h, the media was removed, the staining solution (1:2000 dilution in PBS) was added (500 µL/well), and the plates were incubated, protected from light, at room temperature, for 10 min. Finally, the cells were washed with PBS, photographed using Cytation 1 (BioTek Instruments Inc., Winooski, VT, USA), and analyzed using Gen5™ Microplate Data Collection and Analysis Software (BioTek Instruments Inc., Winooski, VT, USA).

### 2.5. Immunofluorescence

For the immunofluorescent visualization of the cellular components, A375 and RPMI-7951 cells (10^5^ cells/well) were seeded in 12-well plates and stimulated with LUP (10, 30, and 50 µM). After 24 h, the cells were washed with ice-cold PBS, fixed with paraformaldehyde 4% for 1 h at room temperature (RT), and permeabilized with a Triton X/PBS 2% for 30 min at RT, followed by a blocking step with 30% FBS in 0.01% Triton X for 1 h. The actin fibers (AF) were highlighted using Alexa Fluor^®^ 555 Phalloidin antibody (Cell Signaling Technology, Danvers, MA, USA) (overnight incubation at 4 °C). For the staining of microtubules (MTs), the cells were incubated overnight with the primary antibody (α-tubulin antibody, Invitrogen by Thermo Fisher Scientific) in a dark humidity chamber at 4 °C, followed by an incubation (dark humidity chamber, 4 °C, 1 h) with a fluorescent-labeled (Alexa FlourTM 488, Invitrogen by Thermo Fisher Scientific) goat anti-rabbit secondary antibody, the next day. The final step consisted of the counterstaining of the cells’ nuclei with 40,6-diamidino-2-phenylindol (DAPI) for 15 min.

### 2.6. Wound Healing Assay

The influence of LUP on the metastatic character of A375 and RPMI-7951 cells was evaluated by applying the wound healing (scratch) assay. In summary, the cells (10^5^ cells/1 mL/well) were cultured in 24-well Corning plates, and an automatic scratch was made in the middle of each well using the AutoScratch™ Wound Making Tool provided by BioTek^®^ Instruments Inc., Winooski, VT, USA as recommended by the manufacturer. The cells were treated with the test compound at three concentrations (10, 20, and 30 µM) for 24 h and representative images (4× magnification) were taken at the beginning of the assay (0 h) and at its end (24 h) using Cytation 1 and were processed using Gen5 ™ Microplate Data Collection and Analysis Software (BioTek^®^ Instruments Inc., Winooski, VT, USA). The quantification of the effect of LUP in terms of cell migration was performed by calculating the migration rates (%) according to a formula by Felice et al. [21] which has been also used in our previous work [22].

### 2.7. Chorioallantoic Membrane (CAM) Assay

Fertilized chicken eggs were used to determine the potential anti-angiogenic effect. Their preparation consisted of disinfecting the eggs with alcohol of 70% concentration, dating them, and placing them in an incubator in a vertical position. On the 3rd day of incubation, a volume of 7 mL of albumen was extracted with a syringe in order to promote the detachment of the chorioallantoic membrane from the inner shell of the egg, so that the blood vessels are easier to observe. On the next day of incubation, the 4th day, a window was cut in the horizontal part of the eggs which was subsequently covered with adhesive tape, and the eggs were reintroduced into the incubator until the beginning of the experiment. The study of angiogenesis began on the 7th day of incubation. To highlight the potential antiangiogenic effect of LUP, it was tested at a concentration of 50 µM and blood vessels were observed for a period of 24 h. During this time, angiogenesis shows a rapid rate of development, similar to that observed in angiogenesis in the tumor process [23].

### 2.8. Statistical Analysis

All data are expressed as means ± SD, the differences being compared by applying the one-way ANOVA analysis followed by Dunett’s multiple comparisons post-test. The used software was GraphPad Prism version 9.2.0 for Windows (GraphPad Software, San Diego, CA, USA). The statistically significant differences between data were labeled with * (* *p* < 0.1; ** *p* < 0.01; *** *p* < 0.001; **** *p* < 0.0001).

## 3. Results

### 3.1. Cellular Viability and Morphology Assessment

**Figure 1:**
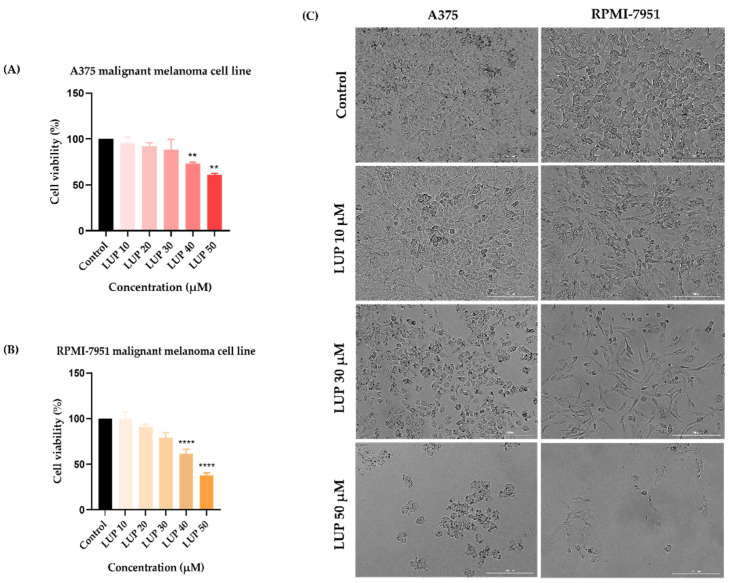
In vitro assessment of the effect LUP (10, 20, 30, 40 and 50 µM) exerts on the viability of (**A**) A375 and (**B**) RPMI-7951 MM cells after 24 h of treatment by applying the MTT assay and (**C**) morphology and confluence of A375 and RPMI-7951 MM cells following the 24 h treatment with LUP 10, 30, and 50 µM. The data are expressed as viability percentages (%) normalized to control cells and expressed as mean values ± SD of three independent experiments performed in triplicate. To identify the statistical differences between the control and the LUP-treated group, a one-way ANOVA analysis was conducted followed by the Dunett’s multiple comparisons post-test (** *p* < 0.01; **** *p* < 0.0001). The image scale bars indicate 200 µm.

**Figure 2:**
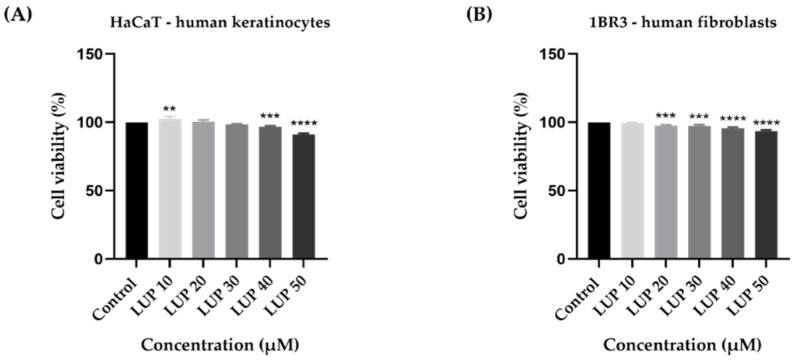
In vitro assessment of the effect LUP (10, 20, 30, 40 and 50 µM) exerts on the viability of (**A**) HaCaT and (**B**) 1BR3 healthy skin cells after 24 h of treatment by applying the MTT assay. The data are expressed as viability percentages (%) normalized to control cells and expressed as mean values ± SD of three independent experiments performed in triplicate. To identify the statistical differences between the control and the LUP-treated group, a one-way ANOVA analysis was conducted followed by the Dunett’s multiple comparisons post-test (** *p* < 0.01; *** *p* < 0.001; **** *p* < 0.0001).

In order to evaluate the potential anti-melanoma effect of LUP, the viability of A375 and RPMI-7951 malignant melanoma cells has been assessed following a 24 h treatment with ascending concentrations (10–50 µM) of LUP. The results indicated a dose-dependent cytotoxic activity of LUP on both cell lines (Figure 1A,B), the highest inhibition of the cell viability being recorded at the highest concentration tested (50 µM) when the percentages reached the values of 61.29% for A375 and 37.78% for RPMI-7951, respectively. Despite the similar viability trend observed in both cell lines, RPMI-7951 cells (IC_50_ = 45.54 ± 1.49) exerted a higher sensitivity to the anti-tumor effect of LUP as compared to A375 (IC_50_ = 66.59 ± 2.20). As a component of the in vitro anti-cancer profile of LUP, its impact on the morphology and confluence of A375 and RPMI-7951 melanoma cells has been examined by monitoring and photographing the cells at the end of the 24 h incubation period. The results confirm the concentration-dependent effect of LUP which initiates the loss of cellular confluency at the lowest concentration of 10 µM. The most prominent effect however has been recorded after the cells’ treatment with the highest concentration of LUP (50 µM). In addition, cell roundness can be observed at 30 and 50 µM which are consistent with cellular death (Figure 1C). The potential skin toxicity has been assessed using human keratinocytes (HaCaT) and fibroblasts (1BR3). After 24 h of treatment, LUP induced a dose-dependent decline in the viability of HaCaT and 1BR3 cells (Figure 2). The highest viability decrease has been registered at the concentration of 50 µM: 91% for HaCaT, and 93.48% for 1BR3 cells. The estimated IC_50_ values are 210.82 ± 25.09 µM (HaCaT), and 346.25 ± 12.84 µM (1BR3).

### 3.2. Nuclear Morphology Evaluation

**Figure 3:**
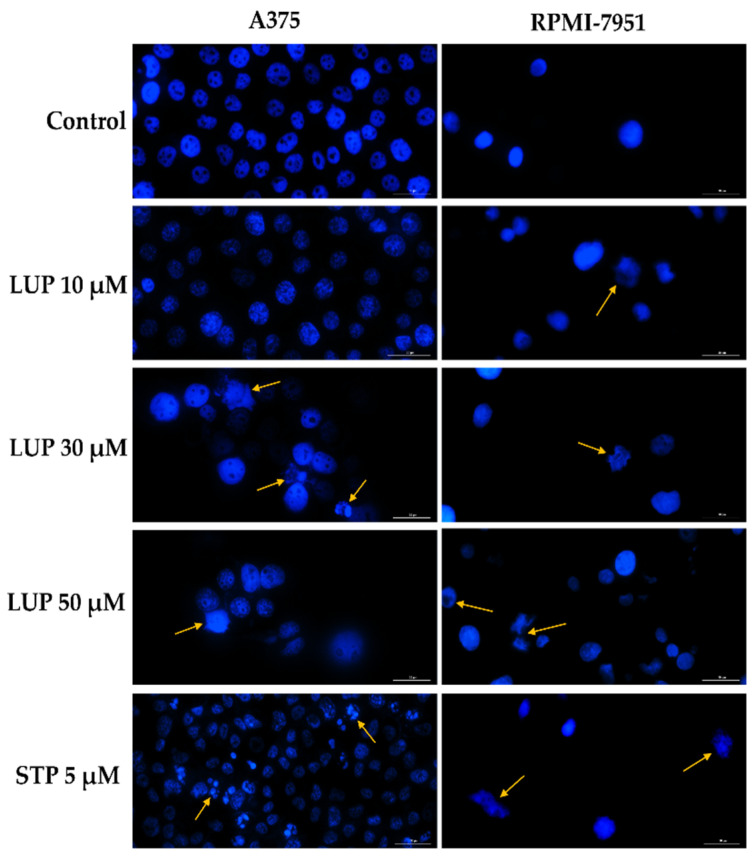
Cell nuclei staining using Hoechst 33342 in A375 and RPMI-7951 malignant melanoma cells following the 24 h treatment with LUP 10, 30, and 50 µM. Staurosporine (STP) 5 µM was selected as positive control for apoptosis. The arrows indicate apoptotic nuclei expressing specific features. The scale bars represent 30 µm.

In order to provide a preliminary evaluation on the cytotoxic mechanism of LUP, the cell nuclei were examined in terms of morphological changes by the means of Hoechst 33342 staining. The interpretation of the results has been performed according to a previous publication by Crowley et al. [24]. Staurosporine (STP) 5 µM was used as positive control for this assay and induced significant changes in the aspect of A375 and RPMI-7951 cellular nuclei, such as fragmentation and dysmorphology. Regarding the effect induced by LUP, in A375 cells no significant changes were detected at 10 µM as compared to the control (untreated cells), while at higher concentrations specific apoptotic features (indicated by arrows) can be noticed, such as nuclear fragmentation, and apoptotic bodies (LUP 20 µM), membrane blebbing, and chromatin condensation (LUP 50 µM). In the case of RPMI-7951 cells, signs of apoptosis (membrane blebbing, fragmentation of cellular nuclei) have been recorded at all concentrations (indicated by arrows). The results are presented in Figure 3.

### 3.3. Immunofluorescence

**Figure 4:**
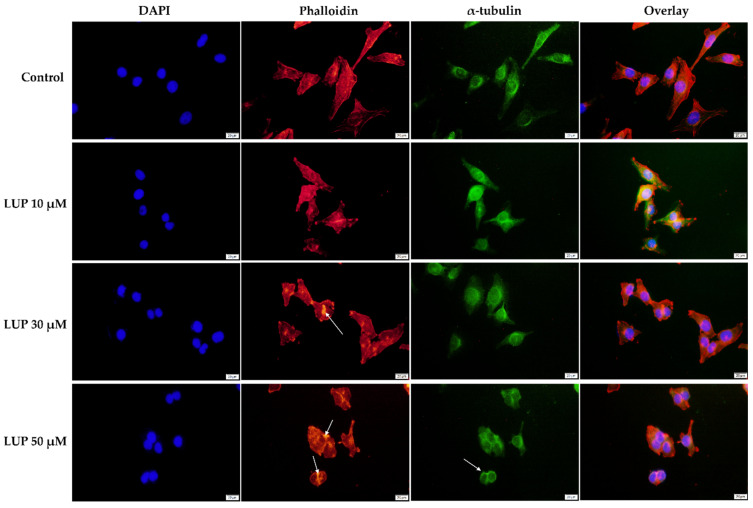
Human melanoma cells (A375) visualized by fluorescence microscopy following the 24 h treatment with LUP (10, 30, and 50 µM). Cell nuclei (DAPI), actin filaments (phalloidin), and microtubules (α-tubulin) are presented individually and also combined (overlay). The scale bars indicate 20 µm.

**Figure 5:**
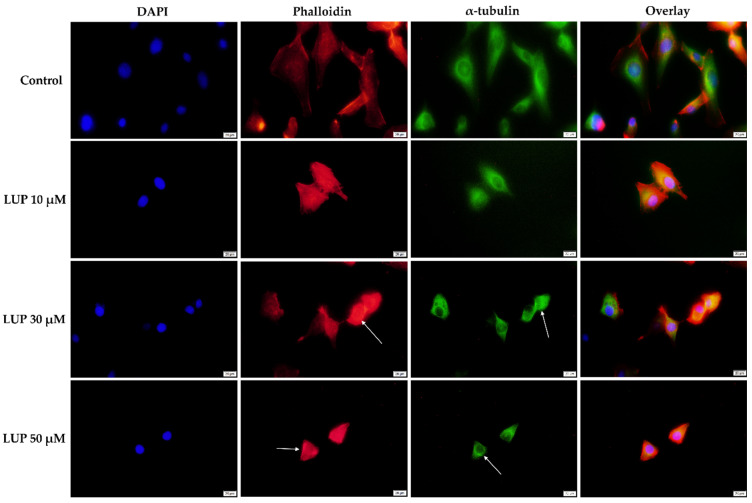
Human melanoma cells (RPMI-7951) visualized by fluorescence microscopy following the 24 h treatment with LUP (10, 30, and 50 µM). Cell nuclei (DAPI), actin filaments (phalloidin), and microtubules (α-tubulin) are presented individually and combined (overlay). The scale bars indicate 20 µm.

To obtain a complete insight into the impact of LUP on the morphology of A375 and RPMI-7951 melanoma cells, a fluorescence immunochemistry technique has been applied highlighting the changes that occurred within the distribution of cytoskeletal components (actin filaments—red, and microtubules—green) following the 24 h treatment. In addition, the cell nuclei were counterstained with DAPI. At the lowest concentration tested (10 µM) LUP induced no significant changes in the aspect of the A375 and RPMI-7951 cells’ cytoskeleton. However, at higher concentrations (30 and 50 µM), several changes in the AF and MTs organization can be observed (indicated by arrows) as follows: (i) in A375 cells (Figure 4)—condensation of actin bundles and reorganization of MTs into a cortical ring leading to cell rounding; and (ii) in RPMI-7951 cells (Figure 5)—actin condensation, perinuclear distribution of MTs, and reduced length of the cells’ longitudinal axis.

### 3.4. Wound Healing Assay

**Figure 6:**
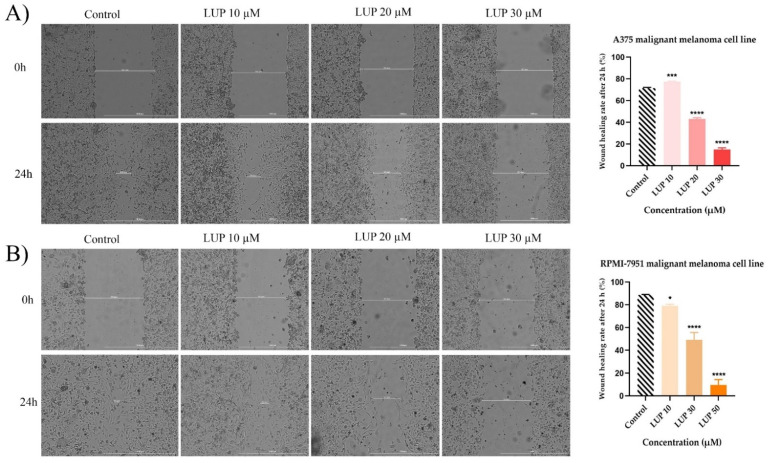
Representative images of the migratory capacity of (**A**) A375 and (**B**) RPMI-7951 MM cells following the treatment with LUP (10, 20, and 30 µM) for 24 h. The scale bars indicate 1000 µm. The bar graphs are presented as wound closure percentage after 24 h compared to the initial surface. The data are expressed as mean values ± SD of three independent experiments performed in triplicate. The statistical differences between the control and the LUP-treated group were identified by applying the one-way ANOVA analysis followed by the Dunett’s multiple comparisons post-test (* *p* < 0.1; *** *p* < 0.001; **** *p* < 0.0001).

Taking into consideration that the high metastatic behavior is a fundamental characteristic of malignant melanoma cells, the potential anti-migratory property of LUP has been investigated by the means of a wound healing assay. For this assessment, the highest concentrations (40 and 50 µM) which caused significant cytotoxicity (Figure 1) in both cell lines were excluded. The effect exerted by LUP on A375 cells (Figure 6A) was highly dependent on the tested concentration. Therefore, at 10 µM (wound healing rate = 77.37%) a slight stimulation of the migratory capacity of cancer cells has been noticed as compared to control (wound healing rate = 71.55%). However, in contrast to this observation, LUP acted as a potent anti-migratory agent at 20 and 30 µM with recorded wound healing rates of 43.15% and 14.98%, respectively. In the case of RPMI-7951 cells (Figure 6B), an augmented effect has been noticed as compared to A375 cells. LUP inhibited the migration of the tumor cells at all concentrations in a dose-dependent manner, the most significant effect being recorded at 30 µM with a registered wound healing rate of 9.66%.

### 3.5. Chorioallantoic Membrane (CAM) Assay

**Figure 7:**
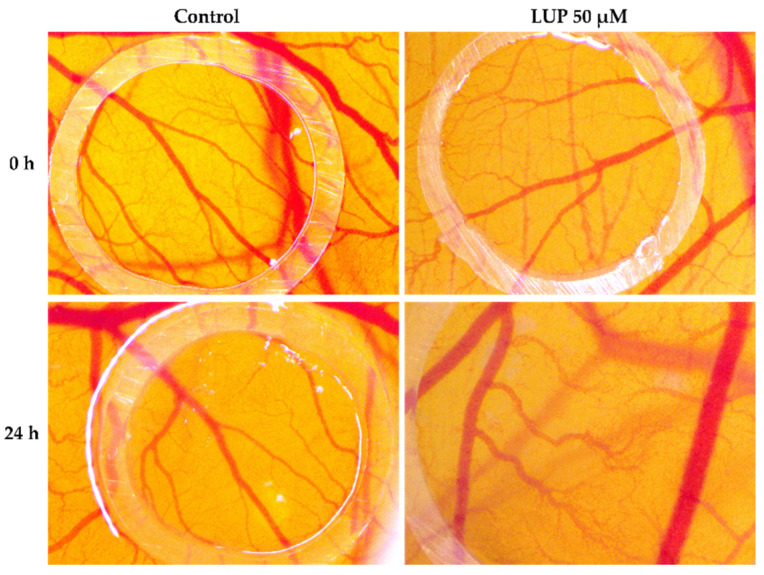
Stereomicroscopic imaging of the chorioallantoic membrane highlighting the angiogenetic process after the treatment for 24 h with LUP 50 µM.

To observe the effect of LUP on blood vessel development, the in ovo method was applied using the CAM assay. The potential antiangiogenic profile of LUP at the highest concentration tested in vitro (50 µM) was examined for 24 h. After this time interval, at the level of the vascular plexus, a series of changes were observed, such as the decrease in the capillary density, with certain areas at the level of the chorioallantoic membrane being devoid of vascularization. In addition, it is noteworthy that the blood vessels formed are devoid of branches, this being a crucial aspect in terms of angiogenesis in the tumor process. Furthermore, at the level of the chorioallantoic membrane, a microhemorrhage can be observed, which after the treatment with LUP was prevented from evolving (Figure 7).

## 4. Discussion

The present study aimed to explore whether LUP acts as a potent anti-cancer dietary compound that might be successfully included in malignant melanoma prophylaxis or chemotherapy, information that is rather inconclusive at present. Accordingly, an in vitro-in ovo investigation of the anti-proliferative, anti-migratory, and anti-angiogenic properties of LUP has been conducted using two MM cell lines—A375 and RPMI-7951. The main findings in this direction reveal that LUP exerts: (i) a dose-dependent and selective cytotoxicity against MM cells at micromolar concentrations (10–50 µM) reflected by a decreased cell viability and confluence, apoptotic nuclear features, cytoskeletal alterations, cellular dysmorphology, and inhibited cell migration (Figure 1, Figure 2, Figure 3, Figure 4, Figure 5 and Figure 6), as well as (ii) a potent anti-angiogenic effect (Figure 7).

LUP is a natural pentacyclic lupane-type triterpenoid [25] displaying a broad spectrum of pharmacological activities, including anti-inflammatory, anti-microbial, anti-diabetic, hepato-, nefro-, and cardio-protective properties which are detailed in an exhaustive review [26]. Furthermore, several studies direct the use of LUP towards the treatment of skin injuries. For instance, Beserra’s research group shows that lupeol exerts beneficial effects in cutaneous repair both in vitro by promoting wound healing in human keratinocytes and fibroblasts, and in vivo by regulating collagen distribution and the expression of several markers (i.e., cytokines, NF-κB, Ki-67, growth factor) [27,28]. Recent research focused on exploring the efficiency of LUP in the treatment of various cancers (i.e., lung, liver, pancreas, head, and neck), offering valuable insight into the molecular mechanisms underlying its anti-tumor properties [29,30,31,32]. With respect to the anti-melanoma effect of LUP, the scientific data remain insufficient. A previous study by Saleem et al. illustrated the ability of lupeol to decrease the viability of WM35 and 451 Lu melanoma cells in a concentration-dependent manner, induce G1-S phase cell cycle arrest, and initiate apoptosis after 72 h of treatment [33]. Similarly, another study by Tarapore and colleagues supports the anti-melanoma effect of LUP against MM cells (Mel 928, Mel 1241, and Mel 1011) by inducing apoptosis and specifically targeting the Wnt/b-catenin signaling after 48 h of treatment [34].

In the light of these previous discoveries, our research focused on gathering supplementary information regarding the anti-melanoma effect of LUP following a short-term treatment (24 h). Our results indicate that LUP decreased the viability of A375 and RPMI-7951 human MM cells in a concentration-dependent trend (Figure 1A,B). The most notable cytotoxic activity has been recorded at the highest concentrations tested (40 and 50 µM), the cellular viability being reduced to 73.17% and 61.29% for A375, and 61.52% and 37.78% for RPMI-7951, respectively. The calculated IC_50_ values were 45.54 ± 1.48 µM for RPMI-7951, and 66.59 ± 2.20 µM for A375, suggesting a cell type-dependent intensity in the anti-melanoma effect of LUP which acted more potently against the RPMI-7951 cell line. As a consequence of the cellular death, a significant loss in cell confluence has been remarked in both cell lines at 30 and 50 µM (Figure 1C). Plant-derived compounds offer substantial benefits in cancer management due to their low toxicity when compared to classical chemotherapeutics [14]. However, toxicological screening is necessary during drug development to ensure the safety of the tested compound. Since in vitro studies represent an eligible platform for revealing the toxicity of substances [35], the viability assay has been performed on human keratinocytes (HaCaT) and fibroblasts (1BR3) as well. The obtained results indicate the absence of LUP-induced cytotoxicity in healthy skin cells in comparison to cancer cells. However, a slight reduction in their viability (to approximately 90%) has been noticed after 24 h of treatment with the highest concentration of LUP (50 µM)—Figure 2. The calculated IC_50_ values—210.82 ± 25.09 µM (HaCaT), and 346.25 ± 12.84 µM (1BR3), are considerably higher than those estimated for A375 and RPMI-7951 MM cells, suggesting a selective anti-melanoma effect.

Apoptosis is a natural mechanism leading to programmed cell death. One of the hallmarks of cancer cells is their ability to evade apoptosis, a process that became a primary target in cancer therapy [36]. The changes generated in the A375 and RPMI-7951 cells’ viability and confluence following the 24 h of treatment led to the implementation of the Hoechst 33342 staining to elucidate the potential cell death mechanism induced by LUP. Nuclear deformation has been observed at 30 and 50 µM in both cell lines, along with apoptosis-specific signs, such as fragmentation, chromatin condensation, membrane blebbing, and formation of apoptotic bodies (Figure 3).

The cytoskeleton is a network formed of intracellular filaments exerting important physiological functions in regard to cancer cell morphology, division, motility, and invasion [37]. During apoptosis, the cell cytoskeleton undergoes a dynamic rearrangement, leading to a change in cell morphology [38,39]. Several cytoskeletal and cell shape alternations have been recorded in A375 and RPMI-7951 MM cells following the 24 h treatment with LUP 30 and 50 µM (Figure 4 and Figure 5), such as condensation of AF, reorganization of MTs into a cortical ring, cell rounding, and reduced longitudinal axis.

Taking into account the invasive nature of malignant melanoma cells, another aspect that has been researched in the present study is the impact of LUP on their migratory ability by performing a wound healing assay. Since at 40 and 50 µM signs of cytotoxicity were detected, the concentrations selected for this experiment were 10, 20 and 30 µM. LUP significantly inhibited the migration of RPMI-7951 cells at all concentrations as compared to the control, with wound healing rates of 79%, 49.21%, and 9.66%. Despite a slight stimulation observed at 10 µM, in A375 cells the results were similar to those obtained in RPMI-7951 cells, the registered wound healing rates being 43.15% and 14.98% at 20 and 30 µM, respectively. The data are presented in Figure 6.

Angiogenesis is a process of real importance in oncogenesis, given that malignant cells need oxygen and nutrients to grow, which are transported through blood vessels. Anti-angiogenic compounds have been developed to inhibit the formation of new blood vessels and also to block tumor growth [40,41,42]. Lupeol raises interest in terms of antiangiogenic effect, noting in the present study that after 24 h of treatment, LUP caused a considerable decrease in neovascularization, and at the chorioallantoic membrane level can be seen areas without vascularization (Figure 7). These results are supported by previous studies in order to highlight the antitumor and antiangiogenic effect of LUP. One such study is the one conducted by Avin et al. [43], in which the antiangiogenic effect of lupeol on the chorioallantoic membrane was similar to that observed in the present study. In addition, by comparing the effect on blood vessels by lupeol and lectin, lupeol has been shown to be more effective due to its small molecular size [44].

## 5. Conclusions

The main objective of the present study was to conduct in vitro and in ovo investigations on the potential of lupeol in the treatment of malignant melanoma. The findings reveal an efficient and selective anti-melanoma effect exerted by LUP at µM concentrations (10–50) against A375 and RPMI-7951 MM cells characterized by: (i) a dose-dependent decrease in the cells’ viability and confluence, (ii) apoptotic nuclear features, (iii) reorganization of the cytoskeletal components, and (iv) inhibition of the cells’ migratory ability. Furthermore, our study suggests that LUP is able to reduce the formation of neovascularization and thus inhibit angiogenesis, being a potential antiangiogenic agent useful in antitumor therapy. Further studies are required for elucidating the mechanisms of LUP-induced in vitro anti-cancer and in ovo anti-angiogenic effects.

## Data Availability

The data presented in this study are available on request from the corresponding author.
